# HOW TO PUBLISH: FROM START TO FINISH AND BEYOND

**DOI:** 10.1093/geroni/igz038.819

**Published:** 2019-11-08

**Authors:** Laura P Sands

**Affiliations:** Virginia Tech, Blacksburg, Virginia, United States

## Abstract

This symposium, organized by the Gerontological Society of America’s (GSA) Publications Committee, will provide information on the publication process from the perspective of several editors of GSA’s scientific journals that publish diverse types of gerontological research, basic to applied across multiple disciplines. This session is comprised of three parts including: 1) Podium presentations from editors-in-chief from GSA’s The Gerontologist, Innovation in Aging (GSA’s new open access journal), and Journal of Gerontology-Series B, Social Sciences. Editors will describe how to prepare your manuscript for submission, choose the right journal, and revise the manuscript for resubmission; 2) A presentation about how to assess and maximize the impact of published work given by a representative from Oxford University Press (OUP); and 3) Round table discussions with editors from the Journals of Gerontology-Series A (Medical Sciences) and B (Psychological and Social Sciences), The Gerontologist, and Innovation in Aging and OUP representatives. Editors will answer questions related to the podium presentations and other questions specific to each journal. Intended audiences include emerging and international scholars, and authors interested in learning more about best practices and tips for getting their scholarly work published. Current and future published authors will also gain information about how to leverage already published work.

